# Psychosocial distress and the quality of life of cancer patients in two health facilities in Cameroon

**DOI:** 10.1186/s12904-022-00981-w

**Published:** 2022-06-01

**Authors:** Bachi-Ayukokang Ebob-Anya, Nahyeni Bassah

**Affiliations:** 1 Department of Nursing, Faculty of Health Sciences, University of Buea, P.O Box 63, Buea, South-West Region Cameroon; 2 Buea Regional Hospital Annex, Buea, South-West Region Cameroon

**Keywords:** Distress, Quality of life, Cancer, Cameroon

## Abstract

**Background:**

Psychosocial distress interferes with the ability to cope effectively with cancer, its physical symptoms and treatment. This in turn leads to poor outcomes in patients.

**Objective:**

The aim of this study was to assess the level of psychosocial distress, emotional distress and the quality of life of cancer patients in two health facilities in Cameroon.

**Methods:**

This study used a cross-sectional hospital-based design. The study was carried out over a period of three months from July–September 2020. The sample size was 120 cancer patients. A consecutive sampling technique was used to select participants. Three validated questionnaires were used: DT, HADS and EORTC QLQ-C30 to assess, psychosocial distress, emotional distress and quality of life respectively. Results were presented using descriptive (frequency, percentage, mean) and inferential statistics (Chi square, Pearson’s correlation, ANOVA). Data were analysed with SPSS version 21. All statistics were considered significant at an alpha value set at 0.05 level.

**Results:**

The majority of patients 83 (69.2%) presented with clinically significant distress, with financial difficulties 87 (72.5%), fatigue 83 (69.2%), transportation 73 (60.8%) and difficulties with work/school 69(57.5%) being the most reported problems. Fifty nine (50.0%) and 56(47.5%) had moderate to severe anxiety and depression symptoms respectively. Overall on HADS, 67 patients (56.8%) presented with emotional distress. The quality of life was fair, with a mean of 52.4 ± 21.3.There was a statistically significant negative relationship (*P* < 0.0001), between psychosocial distress and quality of life of patients.

**Conclusion:**

Cancer patients suffer from psychosocial distress, which has a negative relationship on their quality of life. It is important that healthcare professionals working in these settings, assess psychosocial distress early in patients with cancer to improve the quality of care and enhance quality of life.

**Supplementary Information:**

The online version contains supplementary material available at 10.1186/s12904-022-00981-w.

## Background

Cancer care is rapidly becoming a public health concern in sub-Saharan Africa and Cameroon is no exception [1, 2]. The health-care systems of many sub-Saharan countries are struggling to meet the increasing demand caused by the growing number of patients with cancer, with many services unable to provide adequate care [1]. Despite this, cancer care continues to be given a relatively low public health priority in Africa due to limited resources, lack of awareness and other pressing health problems such as malaria, tuberculosis and HIV/AIDS [3]. The high cancer mortality rates in sub-Saharan Africa, is as a result of poor infrastructure, insufficient numbers of heath care workers, advanced stage presentation, reliance on traditional therapies, few treatment choices and poor compliance [1]. Cancer is not only a physical disease, needing complex and multidisciplinary treatment, but also a very stressful event with significant psychosocial implications related to physical, emotional, spiritual, and interpersonal dimensions [4].Quality of life as defined by WHO is an “individuals’ perception of their position in life in the context of the culture and value systems in which they live and in relation to their goals, expectations, standards and concerns” [5, 6]. This definition by WHO also considers the person’s physical and psychological condition, the level of independence, social relationships, personal beliefs, the environment and culture [5]. To ensure a good quality of life, cancer patients must receive care that is multidimensional and encompasses physical, social, psychological and spiritual domains. Psychosocial distress in cancer patients arises from several factors such as fatigue, pain, anxiety, fear, treatment options, difficulty in transportation, changes in role relationships, physical limitation, fear of recurrence and disappointing social support [7]. Although the link between psychosocial distress and quality of life has been well established in literature, reforms in the healthcare system in Cameroon cannot be made based on information from elsewhere. Therefore, further research and understanding is required on the presence of psychosocial distress in cancer patients, and its relationship to their quality of life in this healthcare context. This research serves as a baseline to further improve the quality of care cancer patients receive in Cameroon.

### Hypothesis

Psychosocial distress has a negative relationship on the quality of life of cancer patients being managed in two health facilities in Cameroon.

## Methods

### Aim

To assess the level of psychosocial distress, emotional distress and the quality of life of cancer patients.

### Study design

This was a descriptive cross-sectional hospital-based study of cancer patients. This design was used because there are no studies in Cameroon that identify the correlation between psychosocial distress and the quality of life of cancer patients, which is needed to guide the development of effective interventions to improve patient care in this group.

### Setting

The study was carried out from 1^st^July 2020 to 30^th^September 2020 at the Douala General Hospital and Cameroon Oncology Centre Bekoko, both in the Economic capital of Cameroon, Douala. The Douala General Hospital is a government owned institution and serves as the largest referral centre for cancer care and treatment in Cameroon. The Cameron Oncology Centre is a new privately owned institution, offering both chemotherapy and radiotherapy services, making it the second largest institution in the nation offering radiotherapy services, after the Douala General Hospital at the moment.

### Sample

The inclusion criteria for the study was patients diagnosed with cancer, who were 18 years and above and gave their consent. Exclusion criteria included, patients in distress or with a serious condition, patients who did not understand English, French or Pidgin and those who had been diagnosed with psychological problems and were receiving specialized psychological treatment for this. A total of 152 patients were approached, with 120 participating in the study, giving a response rate of 78.9%. Consecutive sampling was used to select participants, as all cancer patients were approached and those who met the inclusion criteria were used for the study. This enabled us to work with as many participants as possible to maximise the results of the study.

### Study instruments

Distress was assessed using the National Comprehensive Cancer Network (NCCN) Distress Thermometer (DT), which is self-rated from 0 (no distress) to 10 (extreme distress) and is based on patients’ self-report over the past week. The DT has a cut-off score of 4, with a score of ≥ 4 being indicative of moderate to severe distress. The tool also includes a checklist of 35 practical, physical, family, emotional, and spiritual concerns, which are perceived as causes of distress, and are also self-reported over the same time period [8]. The tool has good validity and reliability and has been extensively used in previous studies to assess distress levels of patients [9–13].

Emotional distress was evaluated using the hospital anxiety and depression scale (HADS). This scale, developed by Zigmond and Snaithin in 1983 [14], is a 14 item self-assessment scale that has been used extensively in previous studies of patients with cancer [15–17]. The HADS tool consists of two sub-scales; an anxiety and a depression scale, each with a total score of 21, which assesses patients’ experiences during the previous week. Its cut-off on an individual scale is ≥ 8, where scores of 8–10 represent moderate symptoms and ≥ 11 represent severe symptoms on either the anxiety or depression section of the scale. Combing both sub-scales gives an overall score on 42, which measures emotional distress with a cut off score of ≥ 15 indicating moderate to severe emotional distress. The HADS scale is not a definitive diagnostic tool for anxiety or depression, rather it serves as an initial tool to identify persons at risk that require further evaluation by healthcare professionals.

The European Organization for Research and Treatment of Cancer Quality of Life Questionnaire (EORTC QLQ-C30) was used to measure quality of life as the primary outcome for the patients living with cancer. It incorporates a global health status, five functional scales (physical, role, cognitive, emotional, and social functioning) and a symptom scales. For the global QOL and functioning scale, higher scores indicate better functioning and for the symptom scale, higher scores indicate higher symptom burden. In general, total scores range from 1–100 and measures the quality of life of patients during the past week [16, 18–21].

### Data analysis

Data were entered into Epi data version 3.1 and analysed with SPSS Version 21. The data were analysed using both descriptive (frequency count, percentage and mean, standard deviation (SD)) and inferential statistics (chi square, Pearson’s correlation, one-way ANOVA). All statistics were considered significant at alpha level 0.05.

### Ethical consideration

Administrative approval was obtained from the Faculty of Health Sciences, University of Buea. Ethical approval was gained from the Faculty of Health Sciences Institutional Review Board (2020/1178–03/UB/SG/IRB/FHS). These were used to gain access to the study hospitals and once all administrative formalities were settled, participant recruitment began. Data collection commenced once consent was gained from individual participants. Participants’ were informed that they were free to withdraw at any time without their care being affected.

## Results

The mean age of patients was 46.1 ± 13.1 years, with values ranging from 18–73 years. The majority of the patients were female 92 (76.7%) and married 69 (57.5%). The most frequent occurring cancers were breast 50 (41.7%) and urogenital 39 (32.5%) (Table 1). Urogenital cancers included cervical, prostrate and urethral cancers. Thirteen (10.8%) were at stage 1 of their disease, 14 (11.7%) at stage 2, 13 (10.8%) at stage 3, 5 (4.2%) at stage 4 and 75 (62.5%) were not aware of their cancer stage. The majority of the patients had children 105 (87.5%) and 21 (17.5%) had other comorbidities (Table 1).

**Table 1:** Socio-demographic characteristics of patients

**S/N**	**Variables**		**Frequency**	**Percentage**
1	Gender	Female	92	76.7
		Male	28	23.3
2	Age category	18–30	14	11.7
		31–50	56	46.7
		> 50	50	41.7
3	Marital status	Single	41	32.4
		Married	69	57.5
		Widowed/divorced	10	8.3
4	Level of education	Primary	23	19.2
		Secondary	56	46.7
		Tertiary	39	32.5
		None	2	1.7
5	Employment status	Employed	71	59.2
		Unemployed	42	35.0
		Retired	7	5.8
6	Monthly income	< 178.70$	20	16.7
		178.70$-893.52$	26	21.7
		> 893.52$	5	4.2
		Nothing	40	33.3
		Varies	12	10.0
		Unanswered	17	14.2
7	Location of cancer	Breast	50	41.7
		Head and neck	19	15.8
		Urogenital	39	32.5
		Gastrointestinal	6	5.0
		Others	6	5.0
8	Mode of treatment	Radiotherapy	15	12.5
		Radiotherapy/Chemotherapy	27	22.5
		Radiotherapy/Surgery	6	5.0
		Radiotherapy/Chemotherapy/Surgery	25	20.8
		Chemotherapy	23	19.2
		Chemotherapy/Surgery	12	10.0
		Surgery	3	2.5
		None	9	6.7

For psychosocial distress, the mean distress level was 4.5 ± 2.7 with values ranging from 0 to 10. The majority of the patients 83 (69.2%) had significant clinical distress as they had a score of ≥ 4, while 37(30.8%) had scores < 4. The most commonly reported problems by participants were; insurance/finance 87 (72.5%), fatigue 83 (69.2%), transport 73 (60.8%), work/school 69 (57.5%), loss of interest in usual activities 67 (55.8%), worry 62 (51.7%), sleep 62 (51.7%), pain 58 (48.3%), appearance 55 (45.8%), waiting time 53 (44.2%) and child care 51 (42.5%). No significant associations were found between socio-demographic factors and the presence of clinically significant psychosocial distress.

The mean anxiety score was 7.7 ± 3.6 with scores ranging from 0–17. Fifty-nine (50.0%) patients had mild, 32 (27.1%) had moderate and 27(22.9%) had severe anxiety symptoms. In total,59 (50.0%) of the patients had scores in the moderate to severe category. On the depression scale, the mean score was 7.6 ± 4.1 with scores ranging from 0–19. Sixty-two (52.5%) had mild, 29(24.6%) had moderate and 27(22.9%) had severe depression symptoms. In total, 56 (47.5%) of the patients had scores in the moderate to severe category. The mean of the overall scale was 15.4 ± 6.7 with scores ranging from 3–32. The majority of the patients 67(56.8%) had an overall score above the cut off score ≥ 15, while 51(43.2%) were below the cut off score < 15. A statistically significant association was seen between psychosocial distress and anxiety and depression with p0.017 and p0.001 respectively. No significant associations were found between socio-demographic factors and anxiety and depression.

The mean score for the general health status (quality of life) was 52.4 ± 21.3 out of 100, and values ranged from 0–100. On the functional scale, cognitive, physical and emotional functioning had the highest mean scores 78.9 ± 23.0, 73.4 ± 23.5 and 72.9 ± 23.6 respectively out of 100. Cognitive functioning, included difficulties remembering and concentrating while reading or watching television. Physical functioning included difficulty doing strenuous activity, walking, being bed bound and needing support with toileting. Emotional functioning included feeling tense, worried, irritable and depressed. Social functioning had the lowest mean value 49.0 ± 38.0, which included participating in family life and social activities. On the symptom scale, financial difficulties had the highest mean score 69.2 ± 36.4, followed by fatigue 39.1 ± 26.3 (Table 2).

**Table 2:** Mean and standard deviation of *EORTC QLQ-C30

S/N	Domain	Mean	*SD	Minimum	Maximum
**Global health status**					
1	Global health status/*QOL	52.4	21.3	0	100
**Functional scale**					
2	Physical functioning	73.4	23.4	7	100
3	Role functioning	59.6	37.9	0	100
4	Emotional functioning	72.9	23.6	0	100
5	Cognitive functioning	78.9	23.0	0	100
6	Social functioning	49.0	38.0	0	100
**Symptom scale**					
7	Fatigue	39.1	26.3	0	100
8	Nausea and vomiting	12.9	20.2	0	100
9	Pain	36.8	35.4	0	100
10	Dyspnoea	17.8	25.2	0	100
11	Insomnia	34.4	37.9	0	100
12	Appetite loss	21.7	31.9	0	100
13	Constipation	16.9	28.0	0	100
14	Diarrhoea	6.1	15.6	0	67
15	Financial difficulties	69.2	36.4	0	100

^*^EORTC QLQ-C30 European Organization for Research and Treatment of Cancer Quality of Life Questionnaire Version 30, *SD* Standard Deviation, *QOL* Quality of Life

One-way ANOVA (Table 3) was used to identify associations between distress levels and the various domains of quality of life. Anxiety was found to have a statistically significant association with emotional functioning,(*P* < 0.0001), and quality of life(*P* < 0.05). On the symptom scale, pain, insomnia and financial difficulties, where also found to be statistically significant(*P* < 0.05).

**Table 3:** Factors Associated With Quality Of Life of Patients

S/N	Domain	*HADS (A)		*HADS (D)		*HADS (overall)		Psychosocial distress	
		**F**	**P**	**F**	**P**	**F**	**P**	**F**	**P**
**Global health status**									
1	Global health status/*QOL	1.985	**0.021**	3.212	**0.000**	3.226	**0.000**	4.060	**0.000**
**Functional scale**									
2	Physical functioning	1.104	0.362	2.712	**0.001**	1.570	0.060	3.100	**0.002**
3	Role functioning	1.613	0.079	2.317	**0.004**	1.098	0.360	2.529	**0.009**
4	Emotional functioning	5.511	**0.000**	2.079	**0.011**	2.702	**0.000**	3.000	**0.002**
5	Cognitive functioning	1.278	0.226	1.531	0.092	1.060	0.404	2.722	**0.005**
6	Social functioning	1.450	0.134	1.860	**0.026**	1.482	0.087	2.739	**0.005**
**Symptom scale**									
7	Fatigue	1.515	0.109	1.855	**0.027**	2.150	**0.004**	4.040	**0.000**
8	Nausea and vomiting	1.238	0.253	1.518	0.096	1.473	0.090	1.210	0.293
9	Pain	2.162	**0.011**	1.671	0.055	2.884	**0.000**	1.294	0.243
10	Dyspnoea	0.536	0.922	2.030	**0.013**	1.182	0.275	2.909	0.003
11	Insomnia	1.892	**0.030**	1.913	**0.021**	2.281	**0.002**	2.610	**0.007**
12	Appetite loss	1.377	0.169	1.081	0.382	2.447	**0.001**	1.359	0.209
13	Constipation	1.484	0.121	0.473	0.968	1.750	**0.027**	0.657	0.576
14	Diarrhoea	1.092	0.373	0.663	0.846	0.878	0.639	1.158	0.327
15	Financial difficulties	2.094	**0.014**	1.673	0.054	1.000	0.478	1.960	**0.045**

^*^HADS (A) Hospital Anxiety and Depression Scale (anxiety sub-scale), *HADS* (D) Hospital Anxiety and Depression Scale (depression sub-scale), *HADS* (overall) Hospital Anxiety and Depression Scale (overall total of both sub-scales combined),* QOL* Quality of Life

Depression was seen to a have a statistically significant relationship with quality of life (*P* < 0.0001). On the functional scale, depression was associated with physical, role, emotional and social functioning (*P* < 0.05). On the symptom scale, there was a statistically significant relationship between depression and fatigue, dyspnoea and insomnia (*P* < 0.05).

In relation to emotional distress, a statistically significant association was found with quality of life, emotional functioning and pain (*P* < 0.0001), and fatigue, insomnia, loss of appetite and constipation, (*P* < 0.05).

For psychosocial distress, a statistically significant association was found between quality of life and fatigue (*P* < 0.0001). Psychosocial distress was also associated with all aspects on the functional scale, insomnia and financial difficulties (*P* < 0.05).

As seen in Table 4, there was a strongly significant negative correlation between quality of life and psychosocial distress, depression and emotional distress (*P* < 0.0001). There was also a statistically significant negative correlation between anxiety and quality of life (*P* < 0.05). Given that higher scores on the quality of life scale indicate better quality of life. There was a strong positive correlation between psychosocial and emotional distress (*P* < 0.0001).

**Table 4:** Correlation between Psychosocial and Emotional Distress and Quality Of Life

S/N	Comparison	Correlation coefficient (r)	*P* value
1	*DT vs. *EORTC QLQ-C30	-0.374^**^	**0.000**
2	Anxiety vs. *EORTC QLQ-C30	-0.289^**^	**0.002**
3	Depression vs. *EORTC QLQ-C30	-0.472^**^	**0.000**
4	*HADS (overall) vs. *EORTC QLQ-C30	-0.448^**^	**0.000**
5	*DT vs. *HADS (overall)	0.519^**^	**0.000**

^*^DT Distress Thermometer, *EORTC QLQ-C30* European Organization for Research and Treatment of Cancer Quality of Life Questionnaire Version 30, *HADS* Hospital Anxiety and Depression Scale. ** Correlation is significant at the 0.01 level.

## Discussion

The mean age of the population of this study was lower than those reported in previous studies [10, 15, 16],with most of those taking part being employed, which also differed from previous research [9, 15, 17, 22]. This finding, shows that despite the presence of disease, many participants were still able to hold down their jobs, which could be explained by the demographic being younger and of working age. Similar to previous studies [12, 22], breast cancer was the most frequent occurring cancer. However, the finding that the majority of the patients were unaware of the stage of their cancer differed from other studies [12, 23]. This highlights that information on the stage of their disease is not being given to patients by the healthcare professionals and confirms the authors experience from practice that the sharing of in depth information is not very common in settings in Cameroon. This experience shows that clinicians are more concerned with treating the disease and often disclose just basic information to the patients, while patients are mostly concerned with the end results, with just a few are being inquisitive about the state of their health. In relation to treatment, most patients received combination therapy as method of management of their cancer, which was also the case with a previous study in Morocco by El Fakir et al. [19].

The NCCN guidelines recommend the screening for distress as the sixth vital sign. This is the first step to identifying those who will benefit from further assessment in order to maximize their health and wellbeing [8]. The finding from this study, highlight that more than half of the patients were above the cut off score for significant clinical distress, which indicates that it is a problem which needs further investigation and implementation into clinical practice. This finding was similar to that of Meeker et al. [11]. The prevalence of psychosocial distress in this study however,was higher when compared to previous studies [10, 12, 13, 22]. One explanation of these higher levels of psychosocial distress could be explained by the resource poor context of the facilities in Cameroon. However, this high prevalence could also be explained by the fact that this study considered the cut off score of four as per the NCCN manual, while others considered ≥ 5 as the cut-off point [10, 12, 13, 22] and therefore reducing the proportion of patients who presented with distress.

The most common problems reported by patients included fatigue, worry, sleep, nervousness, pain, fear and financial difficulties [9, 11, 12, 24]. These difficulties were also reported by most of the participants in this study and while this does not set them apart from previous research, financial difficulties was the most common, followed by fatigue in this study. This finding, could be attributed to the fact that treatment of cancer in these settings in Cameroon is financially demanding with all of the healthcare and treatment costs falling to the patients and their families. Despite most of the patients being employed in this study, they still had significant challenges in keeping up with the financial burden that living with cancer in Cameroon presents. This finding in itself would be enough reason for patients to be in distress, with a lot of uncertainties surrounding the continuation or even commencement of treatment because of lack of adequate finances, which is exacerbated by near inexistent health insurance services. Meeker et al, [11] in their study recommended that interventions aimed at improving distress in patients should also focus on financial distress, by helping patients understand and manage financial obligations and develop financial literacy skills. Fatigue on the other hand is a common symptom in cancer patients, especially for those undergoing radiation therapy or chemotherapy [16]. This could explain the high report of fatigue in this study, as almost all the patients were being treated with either chemotherapy or radiation therapy or both. Moreover, cancer management options including methods of diagnosis, disease related specialists and treatment options are quite limited and centrally located. This means people living with cancer in Cameroon often have to travel long distances, with long waiting times to get access to these services. This phenomenon is common in Cameroon and Africa at large and undoubtedly adds to the fatigue that was experienced by participants in this study. These uncertainties surrounding treatment and outcome are also likely to contribute to the high levels of distress that were evidence in this study. Additionally, cultural factors such as the stigmatization of cancer diagnosis as a death sentence, also play a role in the prevalence of psychosocial distress in this vulnerable population.

Emotional distress, which has always been one of the domains of distress commonly associated with the diagnosis of cancer, has been a particular focus for previous studies [15, 16]. Not only is it a cause for concern at the time of diagnosis, but with advancement in treatment modalities and longer survival rates, there is also increase in the emotional needs of the patients across the cancer pathway [15]. Although distress is a normal response to cancer, the emotional aspect may be related to quality of life, adherence to treatment and satisfaction with care [25]. For this reason, cancer has been associated with a reduction in psychological well-being, quality of life, interpersonal relationships and optimism [26]. As a result, early recognition of the emotional needs of patients, decreases the burden of cancer treatment but at the same time improves quality of life [15]. In this study, a high prevalence of emotional distress in patients was seen when compared to other studies [15–17]. This highlights the importance that the emotional health of patients’ needs to be considered as part of their care planning. Patients in emotional distress are at risk of developing psychiatric disorders, which may affect their compliance to treatment and subsequent outcomes. Hence, assessing across all elements of the psychosocial domain is of upmost importance in cancer care. The psychosocial domain of cancer care, is rarely assessed by healthcare personnel in the study setting. Whilst there are some psychologist and psychiatric specialists, they are not evenly distributed and due to the stigmatization of this domain in our setting, their services are under-utilized. The prevalence of emotional distress seen in this study, should highlight the urgent need to healthcare personnel to initiate routine screening for patients, in order to identify causes and promptly initiate interventions to support patients who are experiencing high levels of distress. Given that emotional distress can arise at any time along the illness trajectory, establishing an effective multidisciplinary approach to this, needs to be considered to ensure improvements in the quality of care of this patient group in settings in Cameroon. The presence of psychological distress is a risk factor for noncompliance to treatment [17, 26] hence, its prevention is vital in patient outcome.

This study did not identify any association between socio-demographic factors and the prevalence of psychosocial and emotional distress. With the high prevalence of psychosocial distress, anxiety and depression and no significant associations between socio-demographic factors, all patients in the study setting are vulnerable. However, an association was seen between psychosocial distress and the presence of anxiety and depression symptoms, provides further evidence of the urgent need for ensuring that screening across the psychosocial domain of cancer care is introduced and addressed across the whole treatment pathway in Cameroon.

Assessment of quality of life is a very important factor for holistic care, as it provides an overall status of the patients’ health without interrupting routine clinical care [18]. The mean overall quality of life score in this study was higher than that reported in some previous studies [20, 21], probably because their studies included only breast cancer patients and was carried out in Iran where contextual factors could have influenced participants’ perception of their general health. However, the mean value was lower in comparison to some other studies [16, 18, 19]. This highlights that, more needs to be done with holistic cancer care approaches in Cameroon to improve the quality of life for patients living with cancer. The social domain, unlike modern societies with well-established programs such as support groups, is almost non-existent in this setting. Most patient to patient interaction, exists informally whilst waiting for appointments and treatment, where patients share their experiences and console each other. The experience of the authors shows that, this can be a problematic as the quality of information shared is not validated by a trained expert, leading to a lot of misconceptions among patients, which has the potential to affect treatment modalities.

There was a strong negative correlation between psychosocial distress, anxiety and depression and the quality of life. Thus as distress, anxiety and depression levels increased in patients, their quality of life worsened. This strengthens the findings in seen in the study where the psychosocial domain a negative relationship with quality of life. Thus pointing out the importance of attending to the needs of patients' in this domain. This study shows that there is an urgent need to implement a multidimensional approach to care, that includes psychosocial domains, across the cancer pathway in the centralised treatment settings in Cameroon. Urgent management strategies, designed by healthcare personnel, are needed to ensure that the quality of life is maximised in this group, paying particular attention to the burden of the financial implications which has a significant effect on distress and overall quality of life for those living and being treated for cancer.

## Conclusion

A good majority of the patients in this study presented with psychosocial and emotional distress. The quality of life of patients was seen to be fair, but there was a strong association between the psychosocial distress, emotional distress and quality of life. This is indicative of the need for improvement of the quality of care which patients living with cancer receive from the health care system in Cameroon.

## Supplementary Information

**Additional file 1: Table 1.** Common Problems Faced by Cancer Patient*s*.

**Additional file 2: Table 2.** Factors Associated With Psychosocial Distress.

**Additional file 3: Table 3.** Factors Associated With Anxiety in Patients.

**Additional file 4: Table 4.** Factors Associated With Depression in Patients.

## Data Availability

The dataset used for the current study are available from the corresponding author on reasonable request due to confidentiality of patients’ information.

## Abbreviations

DT: Distress Thermometer
HADS: Hospital Anxiety and Depression Scale
EORTC QLQ-C30: European Organization for Research and Treatment of Cancer Quality of Life Questionnaire Version 30
ANOVA: Analysis of Variance
SPSS: Statistical Package for the Social Sciences
WHO: World Health Organization
NCCN: National Comprehensive Cancer Network
SD: Standard Deviation
