# Supplementary material for: Psychosocial distress and the quality of life of cancer patients in two health facilities in Cameroon
Source: BMC Palliat Care. 2022 Jun 1;21:96. doi: 10.1186/s12904-022-00981-w (PMC9158288; doi:10.1186/s12904-022-00981-w)
Supplement: Supplementary file 1 — Additional file 1: Table 1. Common Problems Faced by Cancer Patients. [file 12904_2022_981_MOESM1_ESM.docx]

| S/N | **Domain** | **Frequency** | **Percentage** |
| --- | --- | --- | --- |
| **1** | **Practical problems** |  |  |
|  | Child care | 51 | 42.5 |
|  | Insurance/finance | 87 | 72.5 |
|  | Transport | 73 | 60.8 |
|  | Work/school | 69 | 57.5 |
| **2** | **Family problems** |  |  |
|  | Family health issues | 40 | 33.3 |
| **3** | **Emotional problems** |  |  |
|  | Depression | 44 | 36.7 |
|  | Fear | 46 | 38.3 |
|  | Nervousness | 43 | 35.8 |
|  | Sadness | 46 | 38.3 |
|  | Worry | 62 | 51.7 |
|  | Loss of interest in daily activities | 67 | 55.8 |
| **4** | **Physical problems** |  |  |
|  | Appearance | 55 | 45.8 |
|  | Bathing/dressing | 43 | 35.8 |
|  | Fatigue | 83 | 69.2 |
|  | Getting around | 42 | 35.0 |
|  | Pain | 58 | 48.3 |
|  | Sleep | 62 | 51.7 |
| **5** | **Others** |  |  |
|  | Corona virus | 36 | 30.0 |
|  | Waiting time | 53 | 44.2 |
|  | Information | 34 | 28.3 |

Additional Table 1: Common Problems Faced by Cancer Patient*s*
