# Supplementary material for: Psychosocial distress and the quality of life of cancer patients in two health facilities in Cameroon
Source: BMC Palliat Care. 2022 Jun 1;21:96. doi: 10.1186/s12904-022-00981-w (PMC9158288; doi:10.1186/s12904-022-00981-w)
Supplement: Supplementary file 2 — Additional file 2: Table 2. Factors Associated With Psychosocial Distress. [file 12904_2022_981_MOESM2_ESM.docx]

Additional Table 2: Factors Associated With Psychosocial Distress

| **Variables** |  | **Psychosocial** | **distress** | **X^2^** | **P** |
| --- | --- | --- | --- | --- | --- |
|  |  | **Normal** | **High** |  |  |
| **Age** | ≤40 | 23 | 46 | 0.476 | 0.490 |
|  | >40 | 14 | 37 |  |  |
| **Gender** | Female | 28 | 64 |  |  |
|  | Male | 9 | 19 | 0.029 | 0.864 |
| **Companionship** | Yes | 21 | 48 | 0.012 | 0.912 |
|  | No | 16 | 35 |  |  |
| **Employment** | Yes | 28 | 57 | 0.607 | 0.436 |
|  | No | 9 | 26 |  |  |
| **Monthly Income** | Fixed | 16 | 36 | 0.000 | 0.989 |
|  | Unfixed | 21 | 47 |  |  |
| **Education** | Below primary | 7 | 18 | 0.119 | 0.730 |
|  | Above primary | 30 | 65 |  |  |
| **Cancer stage** | Known | 17 | 28 | 1.628 | 0.202 |
|  | Unknown | 20 | 55 |  |  |
| **Children** | Yes | 33 | 72 | 0.140 | 0.709 |
|  | No | 4 | 11 |  |  |
| **Comorbidity** | Yes | 7 | 14 | 0.075 | 0.785 |
|  | No | 30 | 69 |  |  |
| **Treatment option** | Monotherapy | 14 | 27 | 0.019 | 0.889 |
|  | Combination therapy | 23 | 47 |  |  |
