# Supplementary material for: Psychosocial distress and the quality of life of cancer patients in two health facilities in Cameroon
Source: BMC Palliat Care. 2022 Jun 1;21:96. doi: 10.1186/s12904-022-00981-w (PMC9158288; doi:10.1186/s12904-022-00981-w)
Supplement: Supplementary file 3 — Additional file 3: Table 3. Factors Associated With Anxiety in Patients. [file 12904_2022_981_MOESM3_ESM.docx]

Additional Table 3: Factors Associated With Anxiety in Patients

| **Variables** | | **HADS (A) score** | | | **X^2^** | **P** |
| --- | --- | --- | --- | --- | --- | --- |
|  |  | **Mild** | **Moderate** | **Severe** |  |  |
| **Psychosocial distress** | Normal | 25 | 7 | 4 | 8.179 | **0.017** |
|  | High | 34 | 25 | 23 |  |  |
| **Gender** | Female | 45 | 26 | 20 | 0.475 | 0.788 |
|  | Male | 14 | 6 | 7 |  |  |
| **Age category** | ≤40 | 30 | 19 | 19 | 2.946 | 0.229 |
|  | >40 | 29 | 13 | 8 |  |  |
| **Companionship** | Yes | 32 | 19 | 17 | 0.632 | 0.729 |
|  | No | 27 | 13 | 10 |  |  |
| **Level of education** | Below Primary | 10 | 9 | 6 | 1.574 | 0.455 |
|  | Above primary | 49 | 23 | 21 |  |  |
| **Employment status** | Employed | 40 | 23 | 20 | 0.400 | 0.819 |
|  | Unemployed | 19 | 9 | 7 |  |  |
| **Monthly income** | Fixed | 27 | 15 | 10 | 0.713 | 0.700 |
|  | Unfixed | 32 | 17 | 17 |  |  |
| **Children** | Yes | 55 | 26 | 22 | 3.743 | 0.154 |
|  | No | 4 | 6 | 5 |  |  |
| **Illness** | Yes | 11 | 6 | 4 | 0.213 | 0.899 |
|  | No | 48 | 26 | 23 |  |  |
| **Cancer stage** | Known | 22 | 12 | 11 | 0.101 | 0.951 |
|  | Unknown | 37 | 20 | 16 |  |  |
| **Treatment option** | Monotherapy | 19 | 13 | 9 |  |  |
|  | Combination therapy | 34 | 18 | 16 | 0.345 | 0.842 |
