# Supplementary material for: Psychosocial distress and the quality of life of cancer patients in two health facilities in Cameroon
Source: BMC Palliat Care. 2022 Jun 1;21:96. doi: 10.1186/s12904-022-00981-w (PMC9158288; doi:10.1186/s12904-022-00981-w)
Supplement: Supplementary file 4 — Additional file 4: Table 4. Factors Associated With Depression in Patients. [file 12904_2022_981_MOESM4_ESM.docx]

Additional Table 4: Factors Associated With Depression in Patients

| **Variables** | | **HADS (D) score** | | | **X^2^** | **P** |
| --- | --- | --- | --- | --- | --- | --- |
|  |  | **Mild** | **Moderate** | **Severe** |  |  |
| **Psychosocial distress** | Normal | 28 | 6 | 2 | 14.394 | **0.001** |
|  | High | 34 | 23 | 25 |  |  |
| **Gender** | Female | 48 | 23 | 20 | 0.224 | 0.894 |
|  | Male | 14 | 6 | 7 |  |  |
| **Age category** | ≤40 | 34 | 15 | 19 | 2.407 | 0.300 |
|  | >40 | 28 | 14 | 8 |  |  |
| **Companionship** | Yes | 41 | 14 | 13 | 3.867 | 0.145 |
|  | No | 21 | 15 | 14 |  |  |
| **Level of education** | Below Primary | 12 | 4 | 9 | 3.460 | 0.177 |
|  | Above primary | 50 | 25 | 18 |  |  |
| **Employment status** | Employed | 42 | 20 | 21 | 0.943 | 0.624 |
|  | Unemployed | 20 | 9 | 6 |  |  |
| **Monthly income** | Fixed | 27 | 13 | 12 | 0.015 | 0.992 |
|  | Unfixed | 35 | 16 | 15 |  |  |
| **Children** | Yes | 54 | 27 | 22 | 1.706 | 0.426 |
|  | No | 8 | 2 | 5 |  |  |
| **Illness** | Yes | 9 | 6 | 6 | 0.983 | 0.612 |
|  | No | 53 | 23 | 21 |  |  |
| **Cancer stage** | Known | 27 | 6 | 12 | 4.967 | 0.083 |
|  | Unknown | 35 | 23 | 15 |  |  |
| **Treatment option** | Monotherapy | 20 | 13 | 8 |  |  |
|  | Combination therapy | 36 | 15 | 17 | 1.349 | 0.509 |
